# Testing spore amyloidity in *Agaricales* under light microscope: the case study of *Tricholoma*

**DOI:** 10.1186/s43008-020-00046-8

**Published:** 2020-11-11

**Authors:** Alfredo Vizzini, Giovanni Consiglio, Ledo Setti

**Affiliations:** 1 Department of Life Sciences and Systems Biology, University of Torino and Institute for Sustainable Plant Protection (IPSP-SS Turin), C.N.R, Viale P.A. Mattioli, 25, I-10125 Torino, Italy; 2 Bologna, Italy; 3 Mantova, Italy

**Keywords:** *Agaricomycetes*, *Basidiomycota*, Iodine, Melzer’s reagent, nrITS sequences, Pre-heating, Taxonomy of *Tricholomataceae*

## Abstract

Although species of the genus *Tricholoma* are currently considered to produce inamyloid spores, a novel standardized method to test sporal amyloidity (which involves heating the sample in Melzer’s reagent) showed evidence that in the tested species of this genus, which belong in all 10 sections currently recognized from Europe, the spores are amyloid. In two species, *T*. *josserandii* and *T. terreum*, the spores are also partly dextrinoid. This result provides strong indication that a positive reaction of the spores in Melzer’s reagent could be a character shared by all genera in *Tricholomataceae* s. str.

## Introduction

It has been known for about 150 years that some ascomycete and basidiomycete sporomata may contain elements which stain grey to blue-black with iodine-containing solutions. Such a staining was termed amyloid reaction, sometimes written as I+ or J+ (the term “amyloid” being derived from the Latin *amyloideus*, i.e. starch-like), because plant starch gives a similar reaction with iodine (starch-reaction) (Bailey and Whelan 1961; Locquin and Langeron 1978; Immel and Lichtenthaler 2000). The blue colour of the stain is due to the amylose component of plant starch (Takahashi and Ono 1972; Bluhm and Zugenmaier 1981; Moulik and Gupta 1984; Murdoch 1992; Immel and Lichtenthaler 2000). The amylose chain forms a helix shape, and iodine (as triiodide anion I_3_^−^) can be bound inside the helix channel. The other component, amylopectin, gives a red-purple colour which is much less intense than the amylose stain (Bailey and Whelan 1961; Shen et al. 2013). The nature of the starch–iodine interaction is extremely complex and still remains imperfectly known (Bluhm and Zugenmaier 1981; Immel and Lichtenthaler 2000; Shen et al. 2013; Du et al. 2014; Okuda et al. 2020).

An overview of the historical use of Melzer’s was provided by Leonard (2006). Iodine was used in Mycology in the mid-1800s (as alcoholic solutions) mainly for studying lichens and asci (entire ascus wall or apical apparatus). The earliest reference to the use of such a bluing reaction in fungi as a character having a systematic significance is a report of the bluing of a cleistothecial marine ascomycete, *Amylocarpus encephaloides* by Currey (1859). Then the Tulasne brothers (1865), Nylander (1865) and Rolland (1887) noted and described iodine bluing in lichens and Ascomycetes at ascus and ascospore level. Boudier (1885, 1905–1910) was the first to describe and illustrate the use of iodine to classify apothecial Ascomycetes. The first reports of iodine solutions used in Basidiomycetes are those of Patouillard (1887), who described a violet coloration with iodine of the spores of *Cyphella vitellina* (now *Aleurodiscus vitellinus*), and of Rolland (1887), who signaled a blue coloration in the stipe trama of *Mycena tenerrima*. At the time, however, such colour changes observed in micro-anatomical elements of Basidiomycetes were not considered to be systematically relevant by the above mentioned authors. As highlighted by Leonard (2006), it is worth noting that the following year Patouillard (1888) published a paper in which he stressed the importance of spore colour in the classification of fungi, even if he did not mention the use of iodine.

The turning point came when Melzer (1924), after studying *Russula* species for many years, developed a method based on the use of an iodine reagent mixed with chloral hydrate to clear and stain microscopical preparations, which made it possible to study the fine details of the sporal ornamentation of *Russula* species. Baral (1987a) believed that Melzer was “probably inspired by Arthur Meyer”, a German botanist, renowned for his studies on chloroplasts, who introduced the use of chloral hydrate/iodine in Botany under the name “Chloraljod” (Meyer 1883). The formula for what became later known as Melzer’s reagent - and which quickly came into standard use in mycology - is as follows: KI - 1.5 g, iodine - 0.5 g, distilled water - 20 g, chloral hydrate - 22 g). The resulting brownish solution stained dark black-blue the *Russula* sporal ornamentations.

With this reagent, Melzer supplied mycologists with an extraordinarily valuable tool, which has now become so widely used that the term Melzer’s solution (or reagent or simply “Melzer’s”) appears in almost all taxonomic works on the higher fungi. At first, with the exception of some papers by Boursier (1925) and Kühner (1926a, b), Melzer’s reagent was only used by *Russula* specialists because it was invaluable in allowing for an in-depth study of the ornamentation of *Russula* spores (Crawshay 1930; Moreau 1930; Malençon 1931; Singer 1932).

However, this new reagent was soon also used for identification purposes in other groups of pale-spored agarics. Gilbert and Kühner (1928) highlighted that the spore wall is amyloid (blue) in some species of *Amanita*, but unreactive (or inamyloid, i.e. remaining pale yellow-brown or clear) in others. Positive iodine reactions of the spore wall were observed by Kühner (1931, 1936a, 1938) in many species of the genus *Mycena* and, by Kühner and Maire (1934), in many other white-spored agarics. Further, Metrod (1932), Kühner and Maire (1934) and Kühner (1936b) discovered that Melzer’s reagent caused a different, purple-red staining in the spore wall of many species of the genus *Lepiota* and in the basidiome hyphae (especially those of the stipe in *Mycena*) of other agarics. Kühner and Maire (1934) and Kühner (1936a, b) used indiscriminately the term “amyloid” for both the blue and the purple-red reaction on elements of the basidiome. The dark red-brown reaction in Melzer’s reagent was later named pseudoamyloidity by Singer (1938, 1951) and some decades later, dextrinoidity by Orton (1960).

Gilbert (1940) used the following modified solution for *Amanita* spores: distilled water 10 ml, KI 0.5 g, iodine 0.05 g, chloral hydrate 10 g. This solution is almost colourless under the microscope, but the *Amanita* spore walls turn dark grey.

Langeron (1945), in the formulation of Melzers’ s reagent included in his book, used 22 g of chloral hydrate instead of Melzer’s 20 g, but there was no discussion as to why he changed the proportions. This change in the proportions of Melzer’s is now known as “Langeron’s modification” (Ainsworth 1961) and was adopted by Singer (1951, 1963, 1975, 1986).

The contributions of further authors to the generalized use of the Melzer’s reagent led to rapid progress in fungal taxonomy. This was not only especially significant in the taxonomy of the pale-spored agarics (Kühner and Romagnesi 1953; Moser 1967, 1978; Kühner 1980, 1984; Singer 1982, 1986; Horak 2005; Bas et al. 1988; Ludwig 2012; Knudsen and Vesterholt 2018), but it was also beneficial to other non-agaricoid fungal groups in *Agaricomycotina* (e.g., Imler 1950; Donk 1964; Miller 1964; Smith and Zeller 1966; Dodd 1972; Jülich and Stalpers 1980; Watling 1980; Jülich 1984; Hjortstam et al. 1988; Stalpers 1996; Ginns 1998; Ryvarden and Melo 2014; Agerer 2018) and *Pezizomycotina* (Kohn and Korf 1975; Nannfeldt 1976; Rossman 1980; Baral 1987a, b, 2007, 2009; Common 1991; Frey 2016).

In some papers (Kohn and Korf 1975; Nannfeldt 1976; Redhead 1977; Baral 1987a, b, 2007, 2009) the importance of using a KOH pretreatment of the sample to enforce amyloid reactions in ascal tips was stressed. Baral (1987a, b, 2007, 2009) highlighted that Lugol’s, a chloral hydrate-free iodine solution developed in 1829 by the French physician J.G.A. Lugol (initially as a cure for tuberculosis, Calissendorff and Falhammar 2017) is more efficient than Melzer’s in inducing amyloid reactions on apical apparatus in asci.

Iodine solutions (Lugol’s and Melzer’s) are known in *Agaricomycetes* to induce amyloid reactions on other structures besides the spores (Agerer 1999) such as, for example, the hypodermium and pileus and lamellar trama of some *Mycena* species (Kühner 1938), the hymenial cystidia of *Amylocystis* (Singer 1944) and many *Tubulicrinis* (Donk 1964), context hyphae of some *Cortinarius* species (sect. *Purpurascentes*, Moser 1961), hyphae of the stipe base in some *Boletus* species (Imler 1950; Singer 1965; Vizzini et al. 2014), elements in the ectomycorrhizae of *Chroogomphus* and *Rhizopogon* (Smith and Zeller 1966; Agerer 1990; Scambler et al. 2018).

The chemical components of Melzer’s reagent have different functions. Chloral hydrate, being a clearing agent, clarifies and improves the transparency of various dark-colored microscopic materials. Iodine is hardly soluble in water, therefore, potassium iodide is used to improve its solubility (thanks to the reaction I_2_ + I^−^ = I_3_^−^); iodine is thought to be the main active staining agent in Melzer’s, even if its mechanism of action in the amyloid reaction is not entirely understood. It is thought to react/interact with starch-like polysaccharides present in the walls of spores and hyphae, but data in this regard are dated and incomplete (Smith 1965; Blackwell et al. 1985; Morton 1986; Leonard 2006). McCracken and Dodd 1971, Dodd and McCracken (1972) and McCracken et al. (1973) noted that fungal “starch” is different from plant starch in that it is not produced in plastids, is not in granular form, is mainly a cell-wall component (rather than an energy source), and is made up of “only short-chained amylose molecules.” They hypothesized that the amylose in the spore cell helped the spore stay viable until conditions were good for germination. Webster and Weber (2007) suggested that the amylose-like substances prevent oxygen from entering the spore, thereby enforcing dormancy by slowing down metabolism, a hypothesis re-proposed by Halbwachs and Bässler (2015) in their review of spore morphology in agarics. Amylose is insoluble in water and also more resistant to digestion compared to other forms of starch (i.e. amylopectin) (Chen 1990; Bertoft 2017), which could then also contribute to a longer spore dormancy.

A completely different cell wall composition should be the basis of dextrinoidity. Blackwell et al. (2001) suggested that the red-brown reaction with Melzer’s reagent does not involve starch or amylose, but is a reaction with glycine betaine, an osmolyte (an organic osmotic solute) which they found in high concentrations in the Basidiomycetes they studied (*Antrodia carbonica*, *Calvatia* sp., *Chlorophyllum molybdites*, *Ganoderma lucidum* and *Laetiporus sulphureus*).

Focusing on the spores of the pale-spored agarics, the amyloid positive reactions obtained with this medium range from pale grey to blue to violet-black (Meixner 1975; Locquin and Langeron 1978; Erb and Matheis 1983; Brunori et al. 1985; Singer 1986; Charbonnel 2004; Basso 2005). The solution can be stored for a long time (Ammirati et al. 1985; Clémençon 2000, 2012; Lecomte 2017). Usually a positive reaction occurs almost immediately; when the reaction is doubtful, leaving the material in solution for 30 min is recommended (Clémençon 2012). The amyloid reaction may not be of the same intensity on the entire surface of a spore (e.g. *Mycena*, *Gloiothele*, H. Clémençon, pers. comm.). Fresh material sometimes gives a slower reaction than dried material. Melzer’s cannot be used in combination with alkali because iodine reacts with hydroxide ions and a cloudy precipitate develops. Consequently, when potassium hydroxide or ammonium are used as a pretreatment, the alkalinity must be first neutralized before adding Melzer’s (Largent et al. 1977; Ammirati et al. 1985).

The amyloidity of spore-wall was used as a distinguishing taxonomical character at family (i.e. *Bondarzewiaceae*, *Russulaceae*), subfamily/tribus (i.e. *Leucopaxilloideae/Leucopaxilleae*), genus (i.e. *Leucopaxillus*, *Xeromphalina*, *Pseudomphalina*), subgenus (i.e. *Amanita*, *Fayodia*), section level (i.e. *Cystoderma*, *Dermoloma*, *Hydropus*, *Mycena*). Quite recently, the genus *Cystodermella* has been segregated from *Cystoderma* for the species with inamyloid spores (Harmaja 2002), a proposition later supported by molecular analysis (Saar et al. 2009). A curious exception concerning amyloid reactions is *Mycena* sect. *Calodontes*, which encompasses both amyloid and inamyloid spore-producing species. In fact, one of its species, *M*. *pearsoniana*, can show either amyloid or inamyloid reactions interchangeably (Harder et al. 2012). Therefore, presence/absence of an amyloid reaction is clearly a homoplastic character and cannot be used in species delimitation in this section.

At present, it is also known that the amyloid reaction of spore ornamentations is a character that may have evolved several times. Compelling evidence for this is offered by *Leucopaxillus* and *Melanoleuca*, genera traditionally included in the tribe *Leucopaxilleae* or subfamily *Leucopaxilloideae* of the *Tricholomataceae* (Singer 1986; Bon 1991) but which recent molecular studies (Matheny et al. 2006; Dentinger et al. 2016) now place in the tricholomatoid clade (suborder *Tricholomatineae*) and in the Pluteoid clade (suborder *Pluteineae*), respectively.

The amyloid reaction of the spores can sometimes be so weak as to be difficult to assess properly [e.g. in *Pseudoclitocybe* (Bigelow 1982); some species of *Mycena* sect. *Calodontes* (Harder et al. 2012); *Pseudolaccaria* (Lavorato et al. 2015); *Musumecia* (Li et al. 2016] and, consequently, there might be some subjectivity on the part of the operator. To avoid the possible distorting consequences of subjectivity in the assessment of this character, a novel standardized method to test sporal amyloidy has recently been used by the present authors (Alvarado et al. 2018). It allowed us to obtain unambiguous results in cases (e.g. in *Pseudoclitocybaceae*, Alvarado et al. 2018) in which the traditional testing method led to results of dubious interpretation. Based on these outcomes, a project was set up to test with this new method amyloid reactions in the spores of *Agaricales* when observed under light microscopy. It was decided to start, in the present paper, with the species of the large genus *Tricholoma*, typified with *Tricholoma flavovirens* (= *T. equestre*), a widely distributed genus of ectomycorrhizal agarics that was traditionally considered to produce inamyloid spores (Kühner and Maire 1934; Bon 1984, 1991; Singer 1986; Riva 1988, 2003; Christensen and Heilmann-Clausen 2013). However, it has recently been shown to belong to a clade (*Tricholomataceae* s. stricto) containing mostly taxa with positive reaction of the spores in Melzer’s reagent (Sánchez-García et al. 2014).

## Materials and methods

### Morphology

**Fig. 1:**
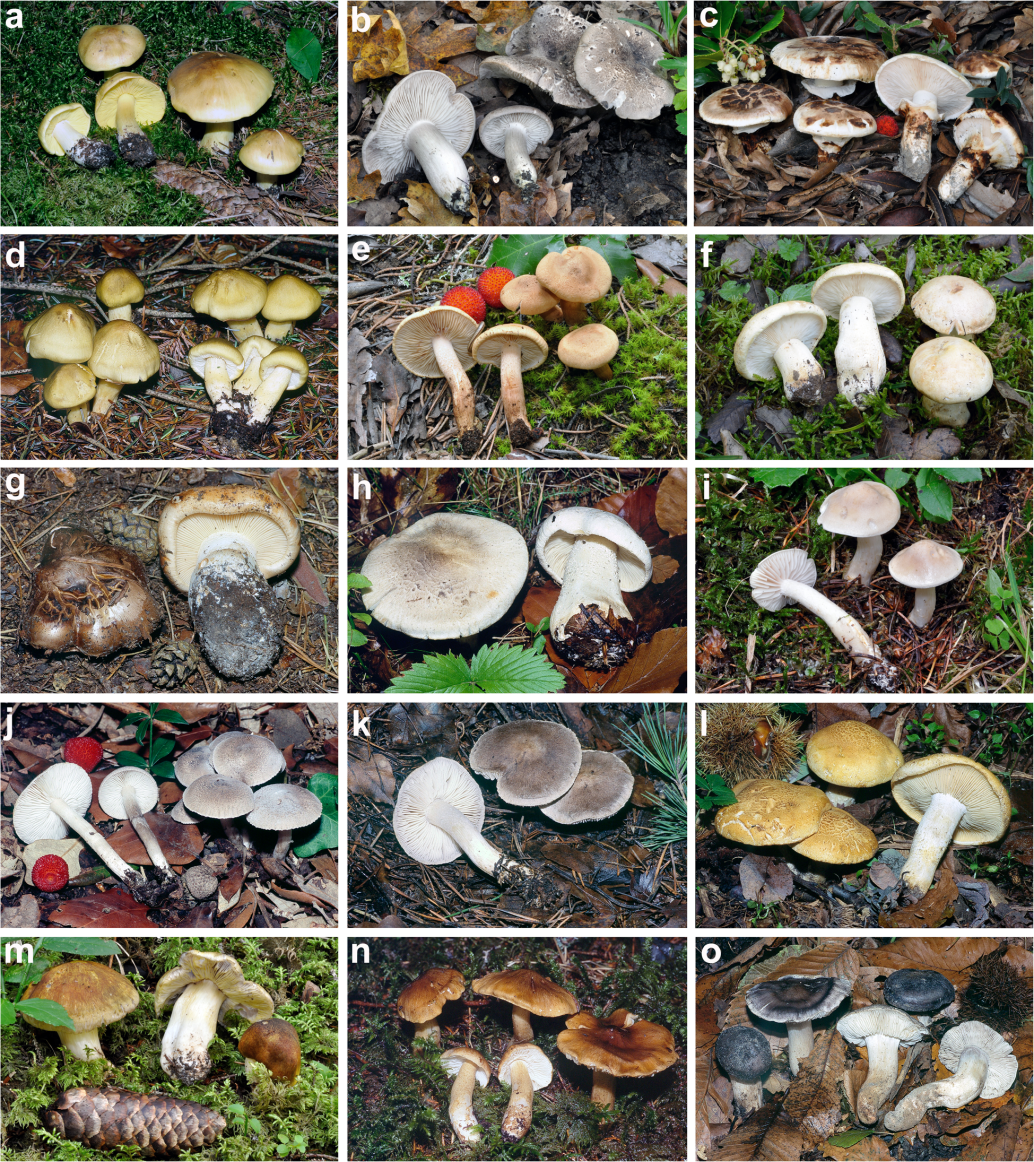
*Tricholoma* collections examined. **a** *T. frondosae* (AMB 17243) **b** *T*. *orirubens* (AMB 17410) **c** *T*. *caligatum* (AMB 17231) **d** *T*. *saponaceum* (AMB 17433) **e** *T*. *psammopus* (AMB 17411) **f** *T*. *acerbum* (AMB 17177) **g** *T*. *colossus* (AMB 17237) **h** *T*. *filamentosum* (AMB 17248) **i** *T*. *inamoenum* (AMB 17367) **j** *T*. *ramentaceum* (AMB 17423) **k** *T*. *terreum* (AMB 17444) **l** *T*. *apium* (AMB 17203) **m** *T*. *arvernense* (AMB 17215) **n** *T. fucatum* (AMB 17300) **o** *T*. *josserandii* (AMB 17407). Photos by G. Consiglio

The eighteen *Tricholoma* collections (corresponding to seventeen species) used for the microscopic analyses (Fig. 1, Table 1) were identified using the monographic work by Christensen and Heilmann-Clausen (2013) and were selected to represent all the ten sections of *Tricholoma* recognized in Christensen and Heilmann-Clausen (2013) and Heilmann-Clausen et al. (2017): *Atrosquamosa*, *Caligata*, *Contextocutis* (= *Saponacea*), *Genuina*, *Lasciva*, *Megatricholoma*, *Pardinicutis*, *Sericella* (= *Sericeocutis*), *Terrea* and *Tricholoma*. Collections of *Tricholoma apium*, *T. arvernense*, *T. fucatum* and *T. josserandii*, species which occupy an isolate position in Heilmann-Clausen et al. (2017), were also studied. Seventeen of these collections have been deposited at AMB and sequenced in the present work, while for one, *T. lascivum* C-F-96230 (shown in Fig. 1 with an asterisk), the nrITS sequence was already present in the public databases GenBank (https://www.ncbi.nlm.nih.gov/genbank/) and UNITE (https://unite.ut.ee/) (LT000028, UDB000005; Heilmann-Clausen et al. 2017). A standardized protocol to test spore amyloidity (named here as the heating method, HM) was applied (used for the first time by the present Authors in Alvarado et al. 2018 and Vizzini et al. 2020): a lamellae sample was hydrated in tap water for 1–2 h or 5% ammonia, and then heated in a drop of Melzer’s reagent (original formulation, Melzer 1924) in a stainless steel spoon until reaching the boiling point at least 1 or 2 times. The sample immersed in Melzer’s was immediately removed from the heat and transferred to a flat Teflon surface, cut into three or four pieces, and transferred again to a slide with a drop of fresh Melzer’s reagent. The excess liquid was then removed and the sample gently squashed and examined with a 60× or 100× bright field objective (numerical aperture NA 1.3, diaphragm open to about 50% range, aperture field 0.9). The classical method (CM) without pre-heating was also used under a different set of conditions: a lamellae sample was hydrated in tap water for 1–2 h or soaked for 3 min. in 5% ammonia, or in 5% KOH, washed and then transferred into Melzer’s, squashed and observed immediately after squashing (CMi), after 30 min. (CM30) and 120 min. (CM120). Furthermore, two species of the genus *Amanita*, *A. phalloides* (subg. *Lepidella*, AMB 18719) and *A. argentea* (subg. *Amanita*, AMB 18720), traditionally recognized as having clearly evidently amyloid and inamyloid spores, respectively (Neville and Poumarat 2004; Tulloss 2020; Tulloss and Possiel 2020), were tested with all the methods (under all the different conditions) above mentioned. These species were chosen as an additional control for the new proposed method. A spore print on glass (microscope slides) obtained from a basidiome of *T. josserandii* (TO AV260920) was tested for amyloidity adding directly a drop of Melzer’s and observed after complete absorption/evaporation of the reagent (2 h) (Fig. 8).

**Table 1:** Spore reaction in Melzer’s depending on the method used

Species	CMi H_2_0/NH_3_/KOH	CM30 H_2_0/NH_3_/KOH	CM120 H_2_0/NH_3_/KOH	HM H_2_0/NH_3_
*Amanita argentea*	inamyloid	inamyloid	inamyloid	inamyloid
*Amanita phalloides*	**amyloid**	**amyloid**	**amyloid**	**amyloid**
*Tricholoma acerbum*	inamyloid	**amyloid**	**amyloid**	**amyloid**
*Tricholoma apium*	inamyloid	**amyloid**	**amyloid**	**amyloid**
*Tricholoma argyraceum*	inamyloid	**amyloid**	**amyloid**	**amyloid**
*Tricholoma arvernense*	inamyloid	**amyloid**	**amyloid**	**amyloid**
*Tricholoma caligatum*	inamyloid	**amyloid**	**amyloid**	**amyloid**
*Tricholoma colossus*	inamyloid	**amyloid**	**amyloid**	**amyloid**
*Tricholoma filamentosum*	inamyloid	**amyloid**	**amyloid**	**amyloid**
*Tricholoma frondosae*	inamyloid	**amyloid**	**amyloid**	**amyloid**
*Tricholoma fucatum*	inamyloid	**amyloid**	**amyloid**	**amyloid**
*Tricholoma inamoenum*	inamyloid	**amyloid**	**amyloid**	**amyloid**
*Tricholoma josserandii*	inamyloid	**dextrinoid/amyloid**	**dextrinoid/amyloid**	**dextrinoid/amyloid**
*Tricholoma lascivum*	inamyloid	**amyloid**	**amyloid**	**amyloid**
*Tricholoma orirubens*	inamyloid	**amyloid**	**amyloid**	**amyloid**
*Tricholoma psammopus*	inamyloid	**amyloid**	**amyloid**	**amyloid**
*Tricholoma ramentaceum*	inamyloid	**amyloid**	**amyloid**	**amyloid**
*Tricholoma saponaceum*	inamyloid	**amyloid**	**amyloid**	**amyloid**
*Tricholoma terreum*	inamyloid	**dextrinoid/amyloid**	**dextrinoid/amyloid**	**dextrinoid/amyloid**

*CMi* spores observed in Melzer’s immediately after squashing and without preheating, *CM30* spores observed after leaving the sample in Melzer’s for 30 min and without preheating, *CM120* spores observed after leaving the sample in Melzer’s for 120 min and without preheating, *HM* spores observed in Melzer’s after heating, *H*_*2*_*0/NH*_*3*_*/KOH* soaking media

Microscopic pictures were taken on a Canon Power Shot A640 digital camera connected to a Zeiss Axioscope 40 microscope with both interferential contrast and phase-contrast optics.

### DNA extraction, amplification and sequencing

Total DNA was extracted from dry specimens (Table 2) employing a modified protocol based on Murray and Thompson (1980). PCR amplification (Mullis and Faloona 1987) included 35 cycles with an annealing temperature of 54 °C. Primers ITS1F and ITS4 (White et al. 1990; Gardes and Bruns 1993) were employed for the region. PCR products were checked in 1% agarose gels, and positive reactions were sequenced with one or both PCR primers. Chromatograms were checked searching for putative reading errors, and these were corrected. The accession numbers of the sequences are reported in Table 2.

**Table 2:** Collections used for the phylogenetic analysis. Newly sequenced collections are in bold

Species	Voucher	Herbarium no.	Country	GenBank accession no.	Unite accession no.
*Hypsizygus marmoreus*	HM561970	–	Malaysia	HM561970	–
***Tricholoma acerbum***	**GC**	**AMB 17177**	**Italy**	**MT462629**	–
*Tricholoma acerbum*	JV99–638	C-F-41483	Denmark	LT000005	UDB001474
*Tricholoma acerbum*	MC00–204	C-F-96223	Slovenia	LT000134	UDB002361
*Tricholoma acerbum*	AF377247	–	Norway	AF377247	–
***Tricholoma apium***	**GC**	**AMB 17203**	**Italy**	**MT462630**	**–**
*Tricholoma apium*	JHC95–049	C-F-35189	Sweden	LT000154	UDB001467
*Tricholoma apium*	JV00–215	C-F-41884	Denmark	LT000009	UDB001685
*Tricholoma apium*	MC98–034	C-F-59207	Norway	LT000118	–
***Tricholoma argyraceum***	**GC**	**AMB 17211**	**Italy**	**MT462631**	–
***Tricholoma argyraceum***	**GC**	**AMB 17441**	**Italy**	**MT462632**	–
*Tricholoma argyraceum*	JHC95–112	C-F-35092	Denmark	LT000010	UDB000780
*Tricholoma argyraceum*	JHC96–244	C-F-96212	Denmark	LT000011	UDB000781
*Tricholoma argyraceum*	JHC97–092	C-F-96213	Sweden	LT000155	UDB000782
*Tricholoma argyraceum*	MEN9491	L0374886 epitype	The Netherlands	LT000198	UDB000785
***Tricholoma arvernense***	**GC**	**AMB 17215**	**Italy**	**MT462633**	–
*Tricholoma arvernense*	MC95–102	C-F-59014	Sweden	LT000157	–
*Tricholoma arvernense*	MC98–020	C-F-59200	Norway	LT000119	UDB002362
*Tricholoma arvernense*	MC98–120	C-F-59255	France	LT000078	UDB001438
***Tricholoma caligatum***	**GC**	**AMB 17231**	**Italy**	**MT462634**	–
*Tricholoma caligatum*	KC565866	–	Algeria	KC565866	–
*Tricholoma caligatum*	JV07–451	C-F-76630	Spain	LT000152	–
*Tricholoma cingulatum*	MC03–252	C-F-96246	Slovakia	LT000128	UDB001420
*Tricholoma cingulatum*	MC96–134	C-F-59057 neotype	Denmark	LT000015	UDB000543
*Tricholoma cingulatum*	MC96–170	C-F-59068	Denmark	LT000016	UDB000544
***Tricholoma colossus***	**GC**	**AMB 17237**	**Italy**	**MT462635**	–
*Tricholoma colossus*	MC01–205	C-F-96238	Slovenia	LT000137	UDB001417
*Tricholoma colossus*	MC97–047	C-F-59154	Sweden	LT000164	UDB001433
*Tricholoma equestre*	MC94–027	C-F-58886	Denmark	LT000018	UDB001508
*Tricholoma equestre*	MC95–187	C-F-96256	Denmark	LT000019	–
*Tricholoma equestre*	MC96–155	C-F-58890	Denmark	LT000020	UDB001469
***Tricholoma filamentosum***	**GC**	**AMB 17248**	**Italy**	**MT462636**	–
*Tricholoma filamentosum*	C-F35924	C-F-35924	Sweden	LT000165	UDB001506
*Tricholoma filamentosum*	JHC0–1202	C-F-96191	Slovenia	LT000138	UDB000804
*Tricholoma filamentosum*	MC00–218	C-F-96226	Slovenia	LT000139	–
*Tricholoma filamentosum*	MC03–242	C-F-96243	Slovakia	LT000129	UDB000803
*Tricholoma frondosae* type I	AF349689	–	USA	AF349689	–
*Tricholoma frondosae* type I	MC95–130	C-F-59031	Sweden	LT000167	–
*Tricholoma frondosae* type I	MC97–151	C-F-59188	Sweden	LT000168	–
***Tricholoma frondosae*** **type II**	**GC**	**AMB 17243**	**Italy**	**MT462637**	–
*Tricholoma frondosae* type II	MC00–225	C-F-96227	Slovenia	LT000140	–
*Tricholoma frondosae* type II	MC96–235	C-F-59084	Denmark	LT000023	UDB001509
*Tricholoma frondosae* type II	MC97–158	C-F-59395	Sweden	LT000169	UDB002363
***Tricholoma fucatum***	**GC**	**AMB 17300**	**Italy**	**MT462638**	–
*Tricholoma fucatum*	MC97–149	C-F-58980 neotype	Sweden	LT000170	–
*Tricholoma fucatum*	MC98–023	C-F-59201	Norway	LT000121	–
***Tricholoma inamoenum***	**GC**	**AMB 17367**	**Italy**	**MT462639**	–
*Tricholoma inamoenum*	JHC95–042	C-F-35182 neotype	Sweden	LT000173	UDB001688
*Tricholoma inamoenum*	MC95–115	C-F-59020	Sweden	LT000174	UDB001424
***Tricholoma josserandii***	**GC**	**AMB 17407**	**Italy**	**MT462640**	–
*Tricholoma josserandii*	MC99–053	C-F-96266	France	LT000081	UDB000797
*Tricholoma josserandii*	MC99–056	C-F-96267	France	LT000082	UDB000798
*Tricholoma lascivum*	JHC03–020	C-F-96194	Slovakia	LT000131	UDB001696
*Tricholoma lascivum*	MC00–519	C-F-96230	Denmark	LT000028	UDB000005
*Tricholoma lascivum*	MC99–197	C-F-59446	Denmark	LT000029	–
***Tricholoma orirubens***	**GC**	**AMB 17410**	**Italy**	**MT462641**	–
*Tricholoma orirubens*	JHC01–200	C-F-96189	Slovenia	LT000141	UDB000524
*Tricholoma orirubens*	JHC93–261	C-F-96208	Denmark	LT000030	UDB000523
*Tricholoma orirubens*	MC03–243	C-F-96244	Slovakia	LT000132	UDB000801
*Tricholoma orirubens*	MC96–301	C-F-59365	Italy	LT000107	UDB000522
*Tricholoma pardinum*	JHC-01201	C-F-96190	Slovenia	LT000142	UDB000802
***Tricholoma psammopus***	**GC**	**AMB 17411**	**Italy**	**MT462642**	**–**
*Tricholoma psammopus*	MC04–600	C-F-96248	Slovenia	LT000145	–
*Tricholoma psammopus*	MC96–345	C-F-59324	Italy	LT000108	–
*Tricholoma psammopus*	MC98–048	C-F-59212	Denmark	LT000036	UDB001472
*Tricholoma psammopus*	MC99–089	C-F-96273	France	LT000084	UDB001503
***Tricholoma ramentaceum***	**GC**	**AMB 17423**	**Italy**	**MT462643**	**–**
*Tricholoma ramentaceum* var. *pseudotriste*	HQ184102	–	France	HQ184102	**–**
***Tricholoma saponaceum***	**GC**	**AMB 17433**	**Italy**	**MT462644**	–
*Tricholoma saponaceum*	C-F23337	C-F-23337	Denmark	LT000038	UDB001499
*Tricholoma saponaceum*	JHC00–049	C-F-96188	Norway	LT000123	UDB001693
*Tricholoma saponaceum*	JHC03–015	C-F-96192	Slovakia	LT000133	UDB001694
*Tricholoma saponaceum*	JHC04–429	C-F-96196	Sweden	LT000185	UDB001697
*Tricholoma scalpturatum*	JHC93–263	C-F-96210	Denmark	LT000042	UDB000541
*Tricholoma scalpturatum*	JHC94–231	C-F-35309	Denmark	LT000043	UDB000542
*Tricholoma scalpturatum*	MC00–207	C-F-96225	Slovenia	LT000146	–
*Tricholoma scalpturatum*	MC95–165	C-F-59399 neotype	Sweden	LT000187	–
***Tricholoma terreum***	**GC**	**AMB 17444**	**Italy**	**MT462645**	–
*Tricholoma terreum*	JHC93–260	C-F-96207	Denmark	LT000057	UDB000536
*Tricholoma terreum*	JHC95–118	C-F-35098	Denmark	LT000058	–
*Tricholoma terreum*	JHC95–172	C-F-35154	Denmark	LT000059	UDB000812
*Tricholoma terreum*	MEN95192	L0374887 neotype	Germany	LT000098	UDB000813

### Phylogenetic analyses

The nrITS dataset was assembled based on that of Heilmann-Clausen et al. (2017). BLASTn (Altschul et al. 1990) was used to select the most closely related sequences from GenBank and UNITE. *Hypsizygus marmoreus* (HM561970) was used as outgroup taxon to root the tree.

Sequences first were aligned in MEGA 6.0 software (Tamura et al. 2013) with its MUSCLE application (Edgar 2004) and then corrected manually. The nrITS alignment was not partitioned into ITS1, 5.8S and ITS2 regions. The Bayesian analysis was performed through the CIPRES Science Gateway platform (Miller et al. 2010) by using the MrBayes v. 3.2.7 algorithm with two simultaneous runs, four chains, temperature fixed at 0.2 and sampling every 1000 generations until reaching the convergence parameters (standard deviation less than 0.01). The first 25% trees were discarded as burn-in. Finally, a full search for the best-scoring Maximum likelihood tree was performed in RAxML v.8.2.10 (Stamatakis 2014) using the standard search algorithm (GTRCAT model, 2000 bootstrap replications). Significance threshold was set ≥0.95 for posterior probability (PP) and ≥ 70% for bootstrap proportions (BP).

## Results

**Fig. 2:**
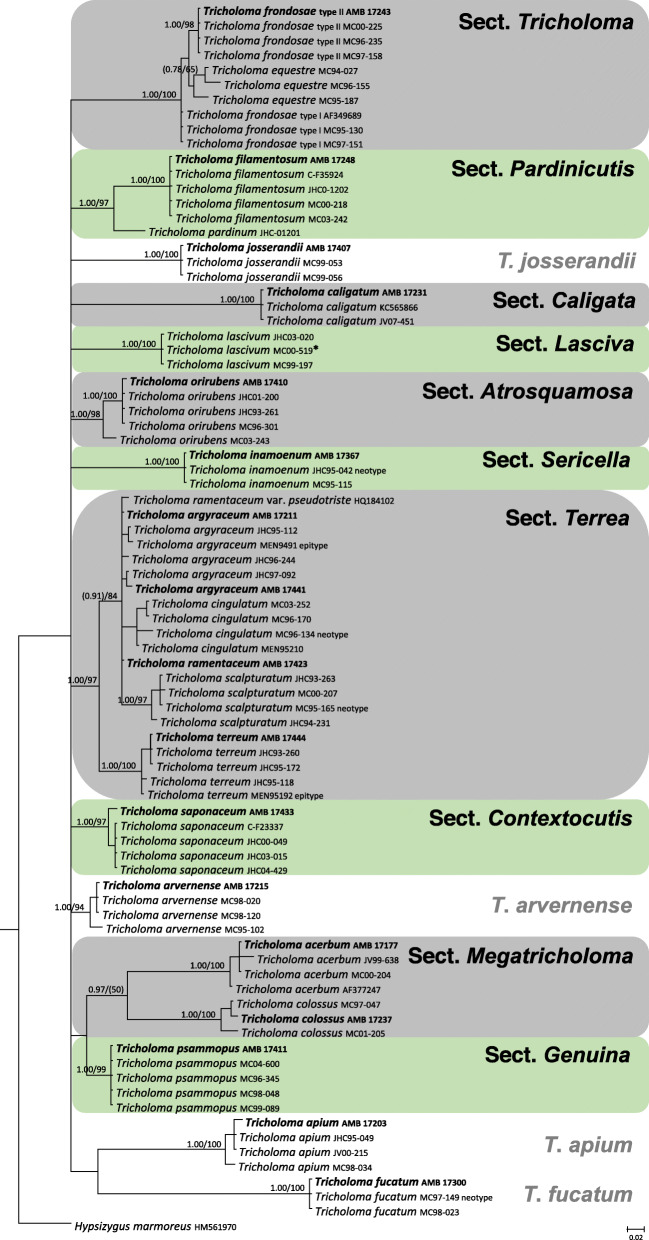
50% majority rule ITS rDNA consensus phylogram of selected *Tricholoma* species (with *Hypsizygus marmoreus* as outgroup) obtained in MrBayes from 114,902 sampled trees. Nodes were annotated if supported by ≥0.95 Bayesian PP (left) and/or ≥ 70% ML BP (right). Non-significant support values are exceptionally represented inside parentheses. Newly sequenced collections used in the morphological analysis are boldfaced. *indicates an additional collection used for testing spore amyloidity

The correct determination of all the *Tricholoma* collections used in this work was confirmed molecularly (Fig. 2). The ten sections (major clades) recognized in Heilmann-Clausen et al. (2017) are also recovered in the present analysis. *Tricholoma apium*, *T. arvernense*, *T. fucatum* and *T. josserandii* are not included in these sections and occupy an isolated position.

**Fig. 3:**
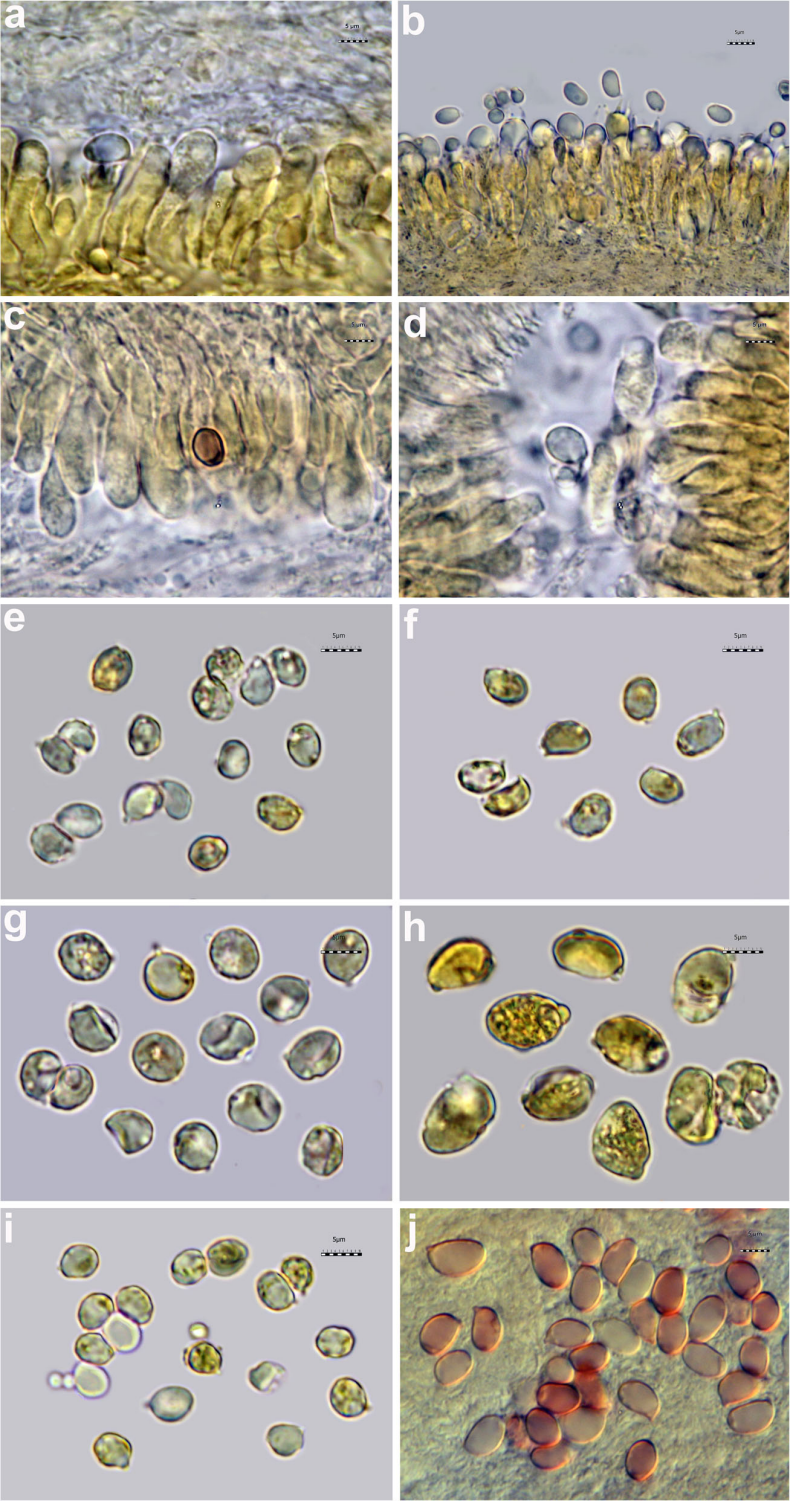
Spores and basidia of *Tricholoma* species observed in Melzer’s after heating. **a–b** *T*. *argyraceum* (AMB 17211) **c–d** *T. josserandii* (AMB 17407) **e** *T*. *caligatum* (AMB 17231) **f** *T*. *frondosae* (AMB 17243) **g** *T. fucatum* (AMB 17300) **h** *T*. *inamoenum* (AMB 17367) **i** *T*. *orirubens* (AMB 17410) **j** *T*. *terreum* (AMB 17444). Photos by L. Setti

**Fig. 4:**
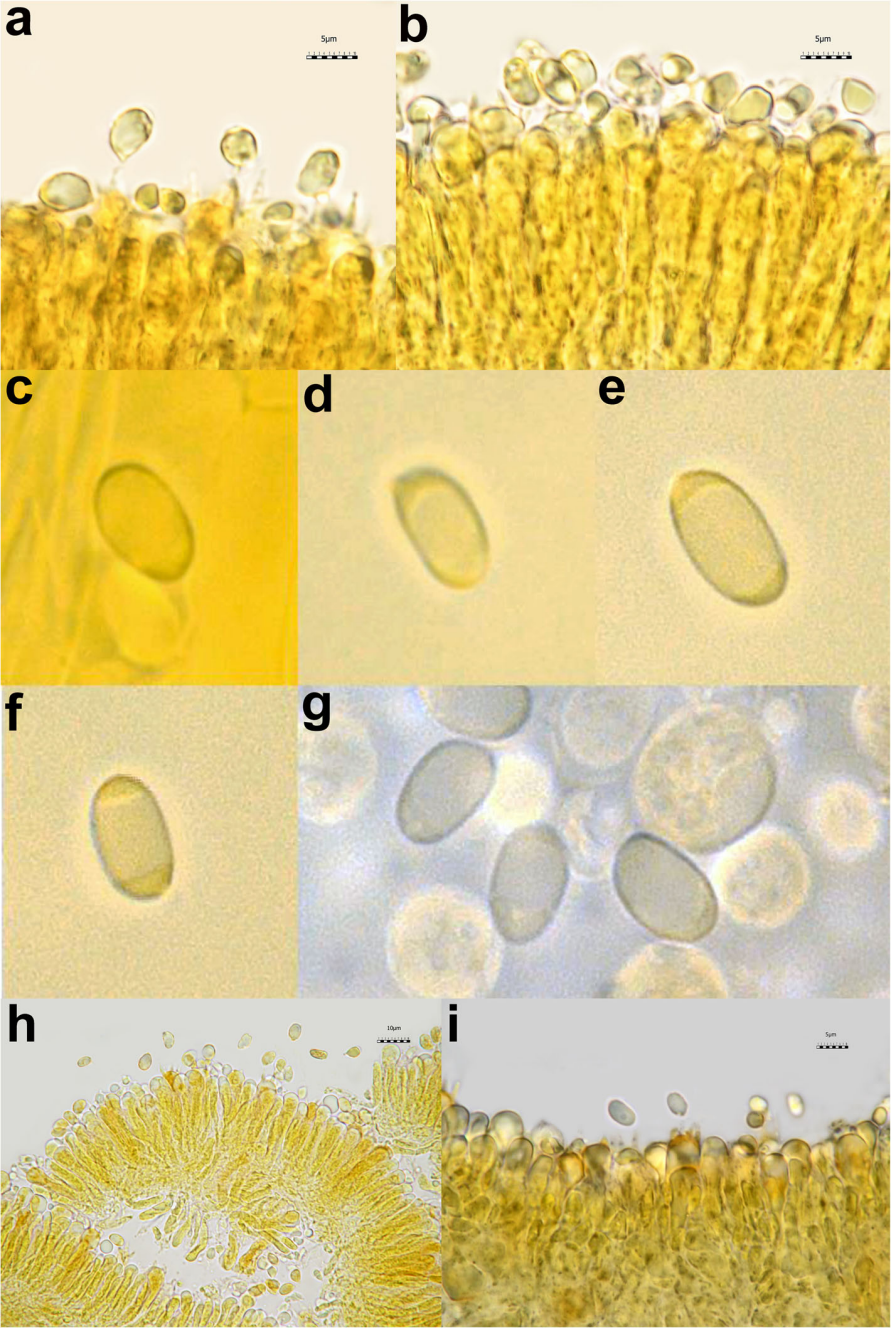
Spores and basidia of *Tricholoma argyraceum* (AMB 17211) observed in Melzer’s without pre-heating. **a**–**b** Sample soaked in 5% ammonia and observed in Melzer’s immediately after squashing. **c–g** Sample soaked in 5% ammonia and observed after leaving the sample in Melzer’s for 30 min. **h**–**i** Sample soaked in tap water and observed leaving the sample in Melzer’s for 30 min. Photos **a**–**b** and **h**–**i** by L. Setti; **c**–**g** by H. Clémençon

**Fig. 5:**
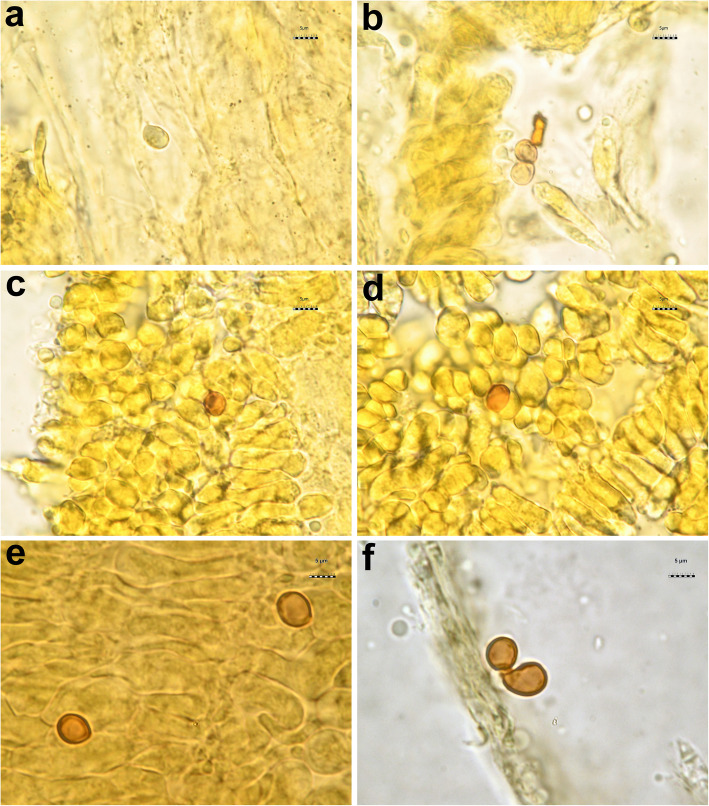
Spores of *Tricholoma josserandii* (AMB 17407). **a**–**d** Observed in Melzer’s without pre-heating. **a**–**b** Sample soaked in 5% ammonia and observed in Melzer’s immediately after squashing; **c**–**d** Sample soaked in 5% ammonia and observed after leaving the sample in Melzer’s for 30 min. **e**–**f** Observed in Melzer’s with pre-heating. Photos by L. Setti

**Fig. 6:**
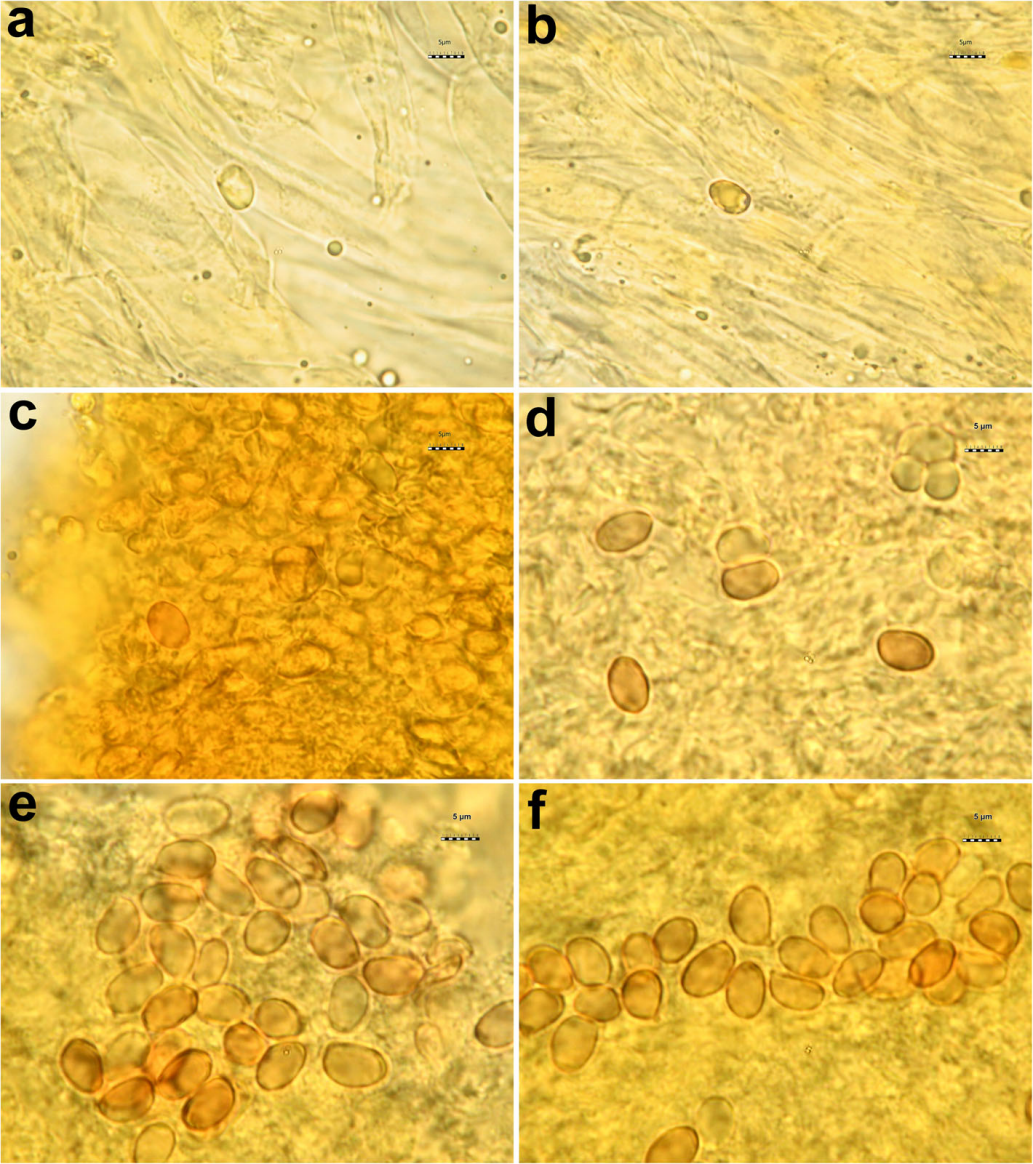
Spores of *Tricholoma terreum* (AMB 17444). **a**–**d** Observed in Melzer’s without pre-heating. **a**–**b** Sample soaked in 5% ammonia and observed in Melzer’s immediately after squashing; **c**–**d** Sample soaked in 5% ammonia and observed after leaving the sample in Melzer’s for 30 min. **e**–**f** Observed in Melzer’s with pre-heating. Photos by L. Setti

All the *Tricholoma* collections showed spores with at least partly dark grey to blue amyloid walls (apiculus included) under the light microscope both by the novel standardized protocol (HM) and the classical method with spores observed after 30 min. (CM30) and 120 m. (CM120), regardless of the soaking medium used (Table 1) (Figs. 3 and 4c–i). The amyloid reaction was also observed on the apical part of basidia (Figs. 3a–d and 4g, i) with both HM and CM30 methods. The classical method with spores observed in Melzer’s immediately after squashing (CMi) always produced negative or difficult to interpret results regardless of the soaking medium used (Table 1) (Figs. 4a–b, 5a–b and 6a–b). Spores of *Tricholoma josserandii* and *T. terreum*, which were negative with the CMi method (Table 1) (Figs. 5a–b and 6a–b) showed an evident dextrinoid reaction (Figs. 5c–f and 6c–f) coupled or not with an amyloid plaque reaction (Fig. 3c–d, j) only with the HM and CM30 methods, regardless of the soaking medium used. The spore print of *T. josserandii* is amyloid (Fig. 8c).

**Fig. 7:**
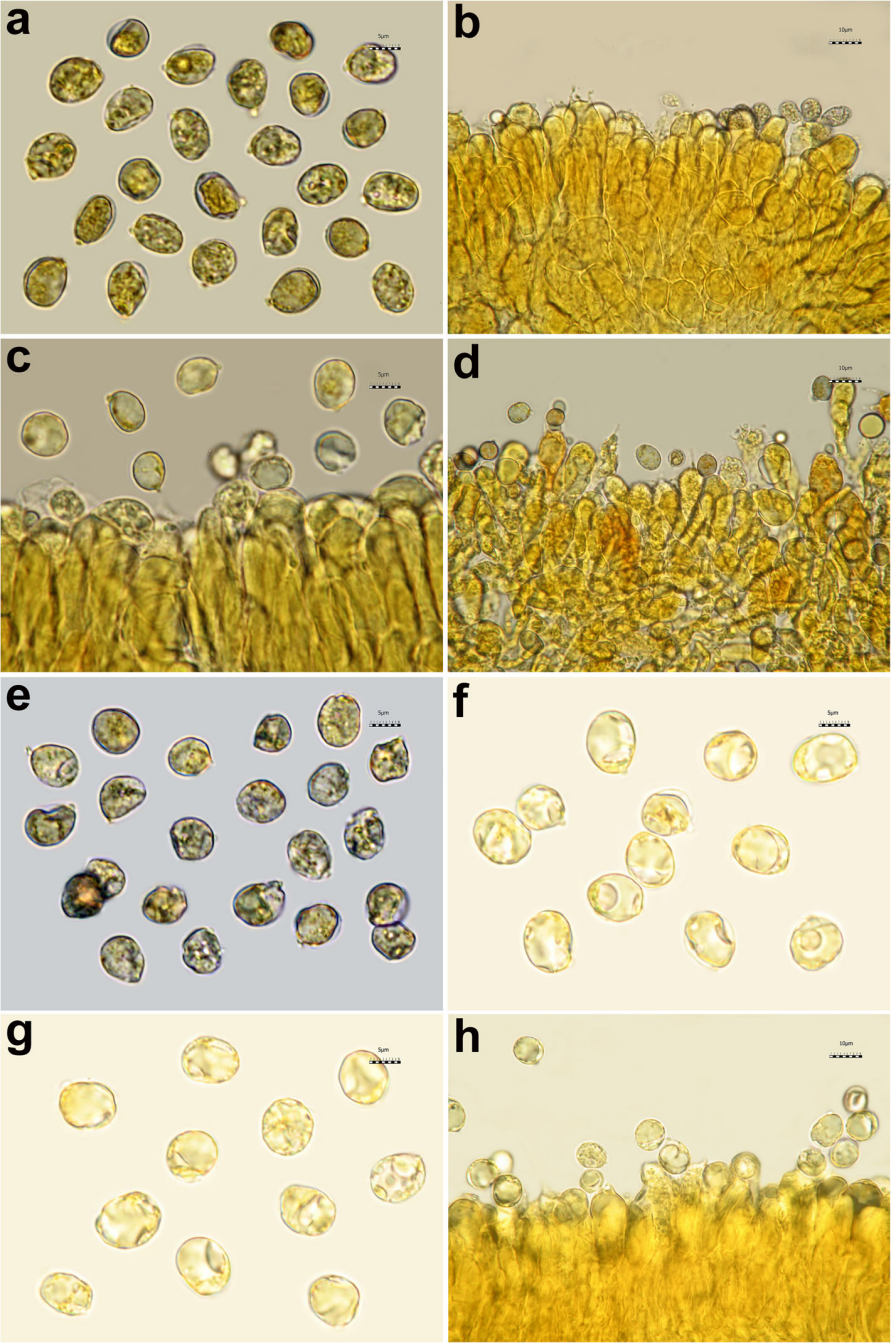
Spores and basidia of *Amanita phalloides* (**a**–**e,** AMB 18719) and *A. argentea* (**f**–**h**, AMB 18720). **a**–**c**, **f** Observed in Melzer’s without pre-heating, immediately after squashing (**a** soaked in tap water, **b**, **f** in 5% ammonia and **c** in 5% potassium hydroxide). **d**–**g** Observed in Melzer’s without pre-heating after leaving the sample in Melzer’s for 30 min (soaked in 5% ammonia). **e**–**h** Observed in Melzer’s with pre-heating. Photos by L. Setti

Spores of *Amanita phalloides* are clearly amyloid (Fig. 7a–e) and spores of *A. argentea* are inamyloid (Fig. 7f–h) by whatever method is used. Some apically amyloid basidia are also present in *A. phalloides* (Fig. 7b–d).

### Specimens examined

*TRICHOLOMA*. **Sect.** ***Atrosquamosa*** – *Tricholoma orirubens*. **Italy**: Emilia-Romagna, Bologna, Lizzano in Belvedere, Vidiciatico, Rio Ri, in a mixed wood of broadleaved trees, 04 Oct. 1993, *G. Consiglio* (AMB 17410). **Sect.** ***Caligata*** *– Tricholoma caligatum*. **Italy**: Lazio, Roma, Santa Marinella, Piano delle Tavole, under *Quercus ilex, Pinus halepensis* and *Arbutus unedo*, 13 Nov. 2015, *G. Consiglio & G. Simonini* (AMB 17231). **Sect.** ***Contextocutis*** – *Tricholoma saponaceum*. **Italy**: Emilia-Romagna, Bologna, Lizzano in Belvedere, Vidiciatico, Rio Ri, under *Fagus sylvatica* and *Abies alba*, 13 Oct. 2001, *G. Consiglio & G. Spisni* (AMB 17433). **Sect.** ***Genuina*** – *Tricholoma psammopus*. **Italy**: Toscana, Siena, Castellina in Chianti, Cipresseta di S. Agnese, under *Cupressus sempervirens* and *Arbutus unedo*, 19 Nov. 2015, *G. Consiglio & E. Franceschini* (AMB 17411). **Sect.** ***Lasciva*** – *Tricholoma lascivum*. **Denmark**: Northern Zealand, Rude Skov, Maglebjerg, under *Fagus sylvatica*, 17 Sept. 2000, *M. Christensen* (C-F 96230, MC00–519). **Sect.** ***Megatricholoma*** – *Tricholoma acerbum*. **Italy**: Emilia-Romagna, Bologna, Monterenzio, Ronchi, in a mixed wood of broadleaved trees, 28 Oct. 1992, *G. Consiglio* (AMB 17177). *Tricholoma colossus*. **Italy**: Trentino, Trento, Baselga di Piné, Laghestèl, under *Pinus sylvestris*, 03 Oct. 2002, *G. Consiglio, G. Marasca & B. Oss-Emer* (AMB 17237). **Sect.** ***Pardinicutis*** – *Tricholoma filamentosum*. **Italy**: Emilia-Romagna, Bologna, Lizzano in Belvedere, Vidiciatico, Rio Ri, under *Fagus sylvatica* and *Abies alba*, 04 Oct. 1993, *G. Consiglio & G. Spisni* (AMB 17248). **Sect.** ***Sericella*** – *Tricholoma inamoenum*. **Italy**: Trentino, Trento, Baselga di Piné, Laghestèl, under *Pinus sylvestris*, 30 Aug. 2007, *G. Consiglio, G. Marasca & B. Oss-Emer* (AMB 17367). **Sect.** ***Terrea*** – *Tricholoma argyraceum*. **Italy**: Marche, Pesaro-Urbino, Piobbico, Monte Grino, in a plantation of *Cedrus atlantica*, *Cedrus deodara*, *Cupressus arizonica* and *Cupressus sempervirens*, 04 Oct. 2007, *G. Consiglio & M. Maletti* (AMB 17211); Lombardia, Mantova, Suzzara, under *Quercus pubescens*, 19 Nov. 2015, *L. Setti* (AMB 17441). *Tricholoma orirubens*. **Italy**: Emilia-Romagna, Bologna, Lizzano in Belvedere, Vidiciatico, Rio Ri, in a mixed wood of broadleaved trees, 04 Oct. 1993, *G. Consiglio* (AMB 17410). *Tricholoma ramentaceum*. **Italy**: Toscana, Firenze, Empoli, La Striscia, under *Quercus ilex* and *Arbutus unedo*, 21 Nov. 2003, *G. Consiglio & E. Franceschini* (AMB 17423). *Tricholoma terreum*. **Italy**: Emilia-Romagna, Bologna, Sasso Marconi, Prati di Mugnano, under *Pinus nigra*, 10 Jan. 1998, *G. Consiglio & G. Spisni* (AMB 17444). **Sect.** ***Tricholoma*** – *Tricholoma frondosae*. **Italy**: Trentino, Trento, Predazzo, Parco di Paneveggio, under *Picea abies*, 24 Sept. 2010, *G. Consiglio & G. Perdisa* (AMB 17243). ***Incertae sedis*** – *Tricholoma apium*. **Italy**: Emilia-Romagna, Bologna, Gaggio Montano, Ronchidoso, in a mixed wood with *Castanea sativa* and *Pinus sylvestris*, 30 Sept. 1998, *G. Consiglio* (AMB 17203). *Tricholoma arvernense*. **Italy**: Trentino, Val di Pejo, Malga Torbi, under *Abies alba*, 30 Aug. 2013, *G. Consiglio, M. Maletti, A. De Angelis & L. Polidori* (AMB 17215). *Tricholoma fucatum*. **Italy**: Trentino, Trento, Predazzo, Bellamonte, under *Picea abies*, 19 Aug. 1998, *G. Consiglio* (AMB 17300). *Tricholoma josserandii*. **Italy**: Emilia-Romagna, Bologna, Grizzana Morandi, Veggio, Tudiano, under *Castanea sativa*, 27 Oct. 1999, *G. Consiglio* (AMB 17407); Piemonte, Torino, Pinerolo, Colle Pra Martino, under *Castanea sativa*, 26 Sept. 2020, *A. Vizzini* (TO AV260920). *AMANITA*. **Subgenus** ***Amanita*** – *Amanita argentea*. **Italy**: Emilia-Romagna, Ronchi (Monterenzio, Bologna), mixed forest with *Quercus pubescens* and *Q. cerris*, 04 July 1994, *G. Consiglio* (AMB 18720). **Subgenus** ***Lepidella*** – *Amanita phalloides*. **Italy**: Trentino, Costasavina (Pergine Valsugana, Trento), mixed deciduous and coniferous forest, 28 Sept. 2005, *G. Consiglio* (AMB 18719).

## Discussion

### Spore amyloidity

**Fig. 8:**
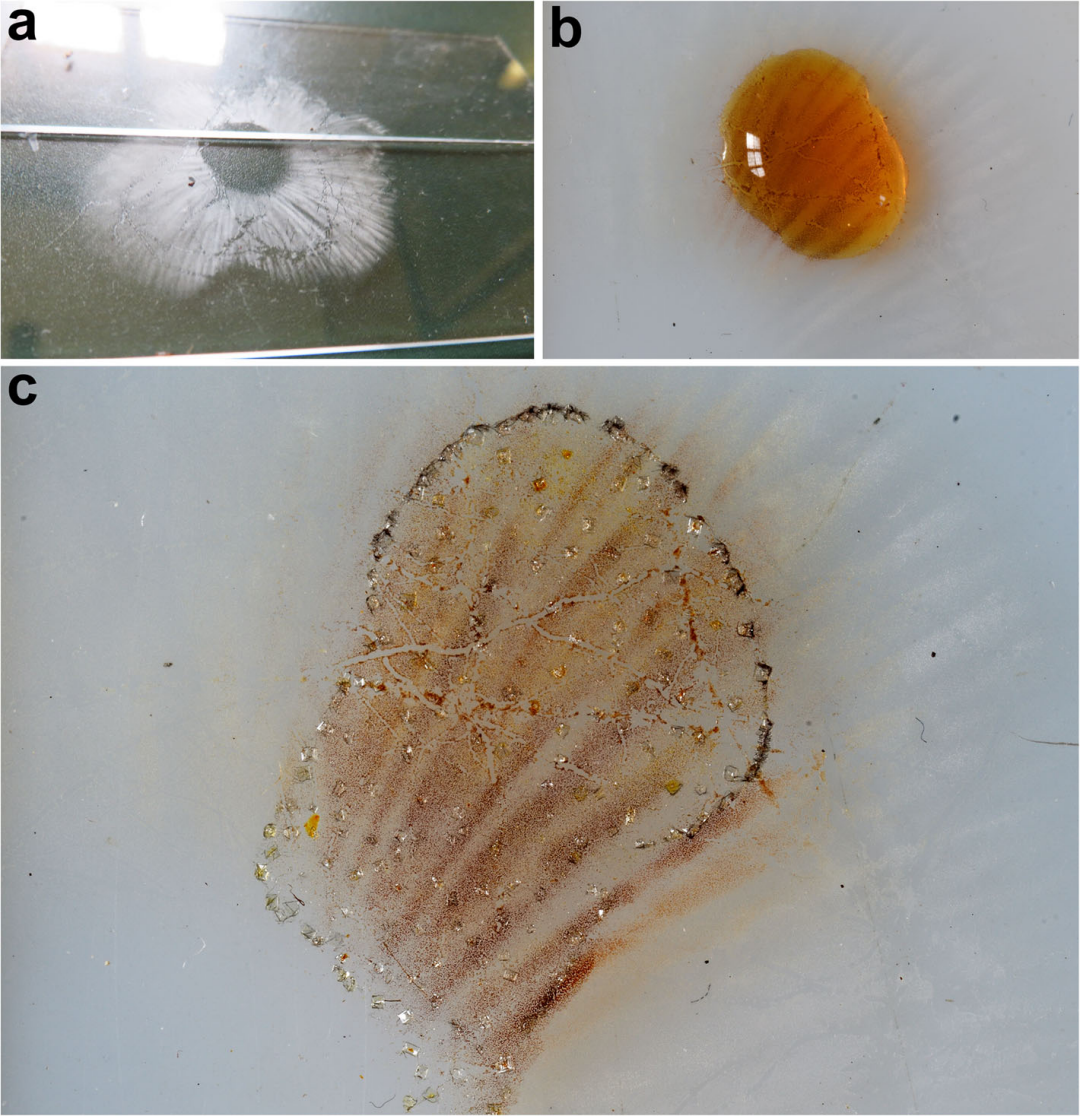
*Tricholoma josserandii* (TO AV260920). **a**. Spore print on microscope slides. **b**. Drop of Melzer’s placed on part of the spore print. **c** Part of the spore print observed after complete absorption/evaporation of the reagent (2 h). Photos by L. Setti

Species identification of the *Tricholoma* collections analyzed, based on pileus colour, structure of the pileipellis, presence/absence of clamp-connections, size and shape of the basidiospores, was supported by molecular data (Fig. 2). All of the eighteen collections studied (seventeen species), which include taxa representative of all the ten sections recognized in *Tricholoma* in Europe by Christensen and Heilmann-Clausen (2013) and Heilmann-Clausen et al. (2017), showed spores with a positive reaction in Melzer’s reagent under light microscope (Figs. 3, 4c–i, 5c–f and 6c–f) by using the pre-heating and the CM30 and CM120 methods (Table 1). The spore print of *T. josserandii* shows an amyloid reaction when treated with a drop of Melzer’s (Fig. 8).

The type of soaking medium used does not appear to have any influence on the iodine reactions of the spore wall, but the use of KOH is not recommended because it can sometimes induce distortions in the shape of some spores; furthermore, if the excess KOH is not well removed, hydroxide ions can react with iodine producing cloudy troublesome precipitates. Instead, very important factors are the preheating or not of the sample and the immersion time of the spores in Melzer’s solution.

With the new method (HM), the amyloid reaction is also evident at the apex of the basidia and sterigmata (Fig. 3a–d). This would seem to indicate structural continuity and an identity of chemical composition between the basidial apex wall and the spore wall. By contrast, the amyloidity test of the same above-mentioned seventeen species, using the classical method (without heating) with spores observed immediately after being mounted in Melzer’s (CMi), produced no clear results (Table 1) (Figs. 4a–b, 5a–b and 6a–b).

However, this is hardly surprising because in the monographic works and in the most important papers dealing with *Tricholoma* worldwide, spores are always reported as inamyloid (Kühner and Maire 1934; Hesler 1958; Horak 1964; Stevenson 1964; Singer (1966); Huijsman 1968; Gulden 1969, 1992; Ovrebo and Tylutki 1975; Bigelow 1979; Ovrebo and Smith 1979; Kost 1981, 1984; Clémençon 1983; Bon 1984, 1991; Ovrebo 1986, 1989; Hongo 1988; Kytövuori 1988; Riva 1988, 1998, 2003; Shanks 1998; Christensen and Noordeloos 1999; Noordeloos and Christensen 1999; Kalamees 2001; Deng and Yao 2005; Galli 2005; Yu et al. 2006; Christensen and Heilmann-Clausen 2008, 2012, 2013; Jargeat et al. 2010; Kirby 2012; Bessette et al. 2013; Hosen et al. 2016; Heilmann-Clausen et al. 2017; Trudell et al. (2017); Yang et al. 2017; Ovrebo and Hughes 2018; Reschke et al. 2018; Ovrebo et al. 2019; Şen and Alli 2019).

As indicated by Clémençon (2000, 2012), pitfalls in assessment of spore amyloidity may be due to several causes, among which first and foremost allowing insufficient time for the reaction to complete. In *Mycena*, he observed that, leaving the sample in Melzer’s for at least 20 min, the spores became black even in species which show very weak reactions (barely discernible under the microscope) when examined in Melzer’s after a short time. Consequently, we tested the eighteen collections also following Clémençon’s advice. First, the samples were soaked for 2 min in 5% ammonia (Fig. 4c–g) or for 2 h in tap water (Fig. 4h–i), then they were transferred to, and squashed in Melzer’s. No amyloid spores could be observed immediately after squashing, but 30 min later some amyloid spores were clearly visible (Fig. 4c–g). Re-observed after an additional 30 min, the number of amyloid spores and the intensity of the amyloid reaction did not change and the same happened after 120 min. Even with the CM30 method some basidia are apically amyloid (Fig. 4g–i).

Interestingly, the spores of *T. josserandii* and *T. terreum* demonstrated a dextrinoid reaction in Melzer’s (Figs. 5c–f and 6c–f), sometimes with small amyloid portions (Fig. 3c–d, j). To our knowledge this situation is absolutely unprecedented in the *Agaricales* and in all *Basidiomycota*.

It is not easy to explain the discrepancy between our results and those of all the other authors who have studied the genus *Tricholoma*. It might be possible that pre-heating the sample in Melzer’s solution somehow allows the starch-like wall material to better interact with the chemical agent and so make the colour reaction much more visible than when it is obtained in Melzer’s at room temperature. The same may happen in unheated samples if the observation is made after waiting the right amount of time (at least 30 min), that is giving iodine the necessary time to get in touch with the amyloid material in the sporal wall.

Whenever it is necessary to highlight the amyloidity of spores of species long known as clearly amyloid (see for example *A. phalloides*, Fig. 7a–e), any method proves effective. But whenever it is intended to analyze the spores of species with known dubious or weak amyloidity (e.g., *Tricholoma* in the present study; *Musumecia*, *Pseudolaccaria*, Alvarado et al. 2018; Vizzini et al. 2020) or of species not yet described or not tested, a negative reaction with the quick classical method (without pre-heating and observation of spores in Melzer’s immediately after squashing, CMi) must be regarded as non-definitive and a stepwise process should be carried out.

In the description of tricholomatoid fungi and in preparing dichotomous keys we would like to suggest to indicate the spores which are amyloid after the CMi method as “immediately amyloid”; after CM30 as “tardily amyloid”; and after HM as “amyloid after pre-heating”.

In conclusion, the authors recommend that the heating method described here, which is as effective as the CM30 but much faster, becomes of common use, and hope that in the future, taxonomic workers will routinely turn to “hot Melzer’s” as a check when determining whether a species which appears to be iodine-negative is truly iodine-negative.

### Phylogenetic implications

*Tricholoma* was first established as a tribus of the broad genus *Agaricus* (Fries 1821). Since then, it has been restricted to ectomycorrhizal fungi with centrally stipitate, fleshy basidiomes with sinuate lamellae, and smooth, hyaline, inamyloid spores (Bon 1984, 1991; Singer 1986). *Tricholoma* is the type genus of the conserved family *Tricholomataceae* R. Heim ex Pouzar (McNeill et al. 2006). *Tricholoma*, the largest genus within the family (about 200 species worldwide according to Kirk et al. 2008), diverged from its saprotrophic sister clades *Dermoloma* and *Pseudotricholoma* during the late Eocene, 62.19 (36.26–92.0) Mya (Sánchez-García and Matheny 2017), and was first demonstrated to be monophyletic by Sánchez-García et al. (2014) based on a multigene analysis. They recovered a major clade, named *Tricholomataceae* s. str., which encompasses seven monophyletic clades corresponding to *Leucopaxillus*, *Tricholoma*, *Pseudotricholoma*, *Porpoloma* s. str., *Dennisiomyces*, *Corneriella*, and *Albomagister*. Sánchez-García et al. (2014) stated that *Tricholoma* and *Albomagister* are the only genera of the family *Tricholomataceae* s. str. with inamyloid spores. A similar phylogeny was included in Sánchez-García and Matheny (2017) and Corriol and Jargeat (2019) who recently reported the first collection of a species of *Dennisiomyces* in Europe. Also *Pseudobaeospora* and *Dermoloma magicum* together with four unidentified *Dermoloma* species were later placed in *Tricholomataceae* s. str. (Desjardin et al. 2014; Sánchez-García and Matheny 2017). *Pseudobaeospora* is considered a genus with clearly dextrinoid spores (Singer 1963, 1986; Bas 2002, 2003; Voto 2009; Arauzo 2011) and *D*. *magicum* show amyloid spores (Arnolds 2002).

In the light of the data reported in the present paper, and considering that in the monospecific genus *Albomagister* has recently included a second species with faintly amyloid spores by Moreau et al. (2015), it seems that a positive reaction of the spores in Melzer’s reagent could be a character shared by all genera in *Tricholomataceae* s. str. and consequently be considered, in this case, a systematic marker at the family level.

## Conclusions

At variance with what has been hitherto believed, the spores of the *Tricholoma* collections sampled here have proved to be amyloid when treated with a Melzer’s solution (original formulation) both with the classical method (without pre-heating), which involves an immersion in Melzer’s for at least 30 min, and a new method proposed in the present work, in which the sample is pre-heated. The soaking media appear to have no influence in inducing the reaction with the Melzer’s solution.

Before being declared inamyloid, the spores of whatever species should be tested by all the methods suggested in this paper. It is hoped that the new method with pre-heating will be generally adopted by mycologists and that it will become widely used. A positive reaction of the spores with Melzer’s reagent appears to be a character present in all genera of *Tricholomataceae* s. str.

## Data Availability

Details of the availability of the data and material used in this study can be found within the text. DNA sequences were submitted to NCBI Genbank database (see Table 2). Dried specimens are deposited in the fungarium listed in the Methods section.

## Abbreviations

5.8S: 5.8S ribosomal RNA
AMB: National Mycological Herbarium of the “Associazione Micologica Bresadola”, Trento, Italy
BI: Bayesian inference
BP: Bootstrap proportions
CM: Classical method for testing spore amyloidity without pre-heating
CMi: Classical method where spores are observed immediately after squashing
CM30: Classical method where spores are observed after 30 min. in Melzer’s solution
CM120: Classical method where spores are observed after 120 min. in Melzer’s solution
CTAB: Cetyltrimethylammonium bromide
DNA: Deoxyribonucleic acid
HM: Heating method, a new method for testing spore amyloidity with pre-heating
ITS1: Internal transcribed spacer 1
ITS2: Internal transcribed spacer 2
Melzer’s: Melzer’s solution/reagent
ML: Maximum likelihood
Mya: Million years ago
nrITS: Nuclear ribosomal internal transcribed spacer
PCR: Polymerase chain reaction
PP: Posterior probability
